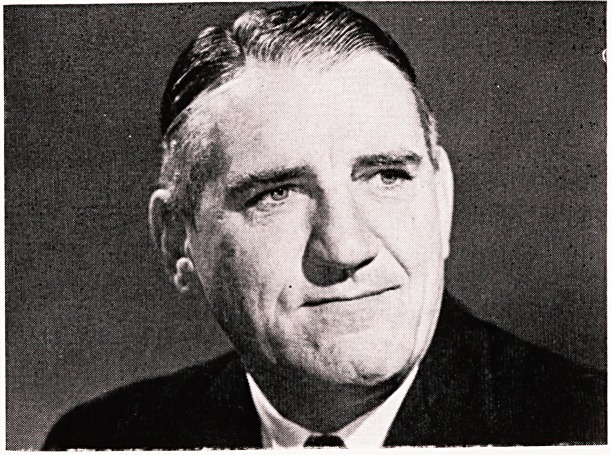# W. M. Capper

**Published:** 1972-01

**Authors:** 


### Obituary

### W. M. CAPPER, M.B., B.S., F.R.C.S., F.R.C.O.G.

Mr. W. M. Capper, Emeritus Consultant Surgeon to the Bristol Royal Hospital and Southmead Hospital, died on December 10th, 1971, after a long illness. He was sixty-three.

He trained at St. Bartholomew's Hospital, qualifying in 1932. He became F.R.C.S. in 1936 and M.R.C.O.G. in 1938. He was elected F.R.C.O.G. in 1939. In addition to his Bristol appointments, he was external examiner in surgery to the Universities of London, Birmingham, Wales and Belfast, and also served on the Court of Examiners of the Royal College of Surgeons. He was a former President of the British Society of Gastroenterology, and also the Christian Medical Fellowship.

William Melville Capper, widely and affectionately known as Bill to most of us, came to Bristol in 1936, succeeding Mr. A. L. Eyre-Brook as Senior Resident Officer at the Bristol Royal Infirmary. Intending to specialise in gynaecology he was in due course appointed Consultant Gynaecologist shortly before the outbreak of the 1939/45 war. During the war years he served in the R.A.M.C. at home and in North Africa with the rank of Lieutenant Colonel in charge of a Surgical Division. After demobilization he returned to Bristol determined to give up gynaecology and start a new career in general surgery. With great courage — typical of the man — he resigned his gynaecological appointment and became a Surgical Registrar. Shortly afterwards there was a vacancy on the surgical staff, and it was quite obvious to everyone who was going to fill that vacancy. Subsequently he was made Clinical Dean, a post he held for several years.

Bill Capper's life and career can be divided into three parts. Firstly his place as a surgeon, secondly his role as a teacher and thirdly and perhaps most important of all, his strong religious beliefs which were largely the guiding factor in his life.

As a gynaecologist turned surgeon after the war he had literally to start from scratch. War and civilian surgery are poles apart and the former is not really a very good framework on which to build the future for general surgery in times of peace. From the beginning he was greatly interested in the surgical treatment of peptic ulceration. Like many of us he became disillusioned with the results of sub-total gastrectomy for duodenal ulcer and from this stemmed his long study and research (with the late Mr. T. J. Butler) on the aetiology of ulceration in relation to the acid and alkali secretions of the gastric mucosa. He was one of the first surgeons in this country to discard the operation of ablation of the stomach for duodenal ulcer in favour of the less radical procedure of vagotomy and pyloroplasty. For a large man, he had a surprisingly good pair of hands and to watch Bill doing a gastric operation with great panache and delicacy was quite a revelation.

He was a magnificent teacher, quite one of the best the Medical School has produced since the days of Professor Rendle Short his old friend and chief. He was lucid and clear, and had the gift of infecting his students with the same boyish enthusiasm that he himself had possessed for so long. A big man with a big heart he loved his students and they responded to him. As Clinical Dean, he was understanding and sympathetic yet firm and unyielding when a matter of principle was involved. Many students over the years must have cause to be grateful to him for his unfailing kindness and guidance to them in the various problems that inevitably beset the students of today.

Bill Capper was a real Christian who practised what he preached. His religion was his whole life. Honest and straightforward, humble and sincere, there was nothing sanctimonious or proselytising about him; he just had a simple faith from which he never wavered. In this, as in all things, he was ably supported and encouraged by his wife May who had always been a tower of strength to him. As Disraeli once said 'Religion should be the rule of life, not a casual incident of it'. This is surely a fitting epitaph for Melville Capper.

All who knew this lovable man will agree that many years must pass before we see his like again.

## Figures and Tables

**Figure f1:**